# Shifting the burden or expanding access to care? Assessing malaria trends following scale-up of community health worker malaria case management and reactive case detection

**DOI:** 10.1186/s12936-017-2088-1

**Published:** 2017-11-02

**Authors:** David A. Larsen, Anna Winters, Sanford Cheelo, Busiku Hamainza, Mulakwa Kamuliwo, John M. Miller, Daniel J. Bridges

**Affiliations:** 1 Department of Public Health, Food Studies and Nutrition, Syracuse University, Syracuse, NY USA; 2 Akros, Lusaka, Zambia; 3 University of Montana School of Public and Community Health Sciences, Missoula, MT USA; 4 National Malaria Elimination Centre, Lusaka, Zambia; 5 A Program at PATH, Malaria Control and Elimination Partnership in Africa, Lusaka, Zambia

**Keywords:** Surveillance, Elimination, Community case management, Reactive case detection

## Abstract

**Background:**

Malaria is a significant burden to health systems and is responsible for a large proportion of outpatient cases at health facilities in endemic regions. The scale-up of community management of malaria and reactive case detection likely affect both malaria cases and outpatient attendance at health facilities. Using health management information data from 2012 to 2013 this article examines health trends before and after the training of volunteer community health workers to test and treat malaria cases in Southern Province, Zambia.

**Results:**

An estimated 50% increase in monthly reported malaria infections was found when community health workers were involved with malaria testing and treating in the community (incidence rate ratio 1.52, p < 0.001). Furthermore, an estimated 6% decrease in outpatient attendance at the health facility was found when community health workers were involved with malaria testing and treating in the community.

**Conclusions:**

These results suggest a large public health benefit to both community case management of malaria and reactive case detection. First, the capacity of the malaria surveillance system to identify malaria infections was increased by nearly one-third. Second, the outpatient attendance at health facilities was modestly decreased. Expanding the capacity of the malaria surveillance programme through systems such as community case management and reactive case detection is an important step toward malaria elimination.

**Electronic supplementary material:**

The online version of this article (10.1186/s12936-017-2088-1) contains supplementary material, which is available to authorized users.

## Background

Malaria accounts for an estimated 16% of child mortalities in sub-Saharan Africa [1]. Inequities in access to treatment can lead to increased morbidity and mortality rates in populations distant from health centres due to further delays in treatment [2, 3]. In Zambia, treatment-seeking behaviour is perhaps the largest barrier to ensuring prompt access to anti-malarials [4]. Expanding malaria treatment services through community case management (CCM) of malaria has shown to be an effective method of improving individual access to malaria treatment [5], but information regarding the impact of CCM on the health system remains understudied.

Long wait times and staff shortages are associated with poor health systems throughout sub-Saharan Africa [6–8], a problem to which malaria contributes directly with its estimated 165 million incident cases in 2013. There are simply too many malaria cases for the number of health workers on the continent [9]. The HIV/AIDS pandemic, outmigration of trained health staff, and a lack of funding to health systems contribute to the problem of an inadequate health system, which in turn leads to challenges in reducing mortality caused by preventable and treatable infectious diseases [10]. This shortage of health workers is felt in Zambia [11], and most prominently in rural areas [12].

Malaria and other illnesses such as pneumonia and diarrheal disease can be safely addressed by community health workers (CHW) with 1–2 week trainings to administer simple diagnostics and medicines [13]. In Zambia, CCM of malaria through CHWs has been implemented in various settings following a policy shift to allow CHWs to conduct malaria rapid diagnostic tests and treat those testing positive with artemether-lumefantrine [14, 15]. Expanding on this policy in Southern, Central and Western Provinces of Zambia, a reactive case detection (RCD) system has been implemented wherein CHWs perform case investigations for incident malaria cases suspected of being locally acquired [16]. RCD is based on the premise that incident malaria may be representative of local malaria transmission, likely in the immediate vicinity of the incident case’s household [17]. While CCM refers to CHW efforts to test and treat symptomatic community members who seek care for their illness, RCD is an additional service whereby CHWs follow-up incident cases to their household, and then test and treat around the incident case. CCM with RCD is increasingly viewed as a key element of the Zambian Government’s national elimination efforts especially in areas in the final hurdle of identifying residual foci and stomping out local remaining infections (Malaria elimination strategy—Zambia 2016). This article assesses the impact of CCM and RCD on the malaria burden at the health system level.

## Methods

### Study site

**Fig. 1:**
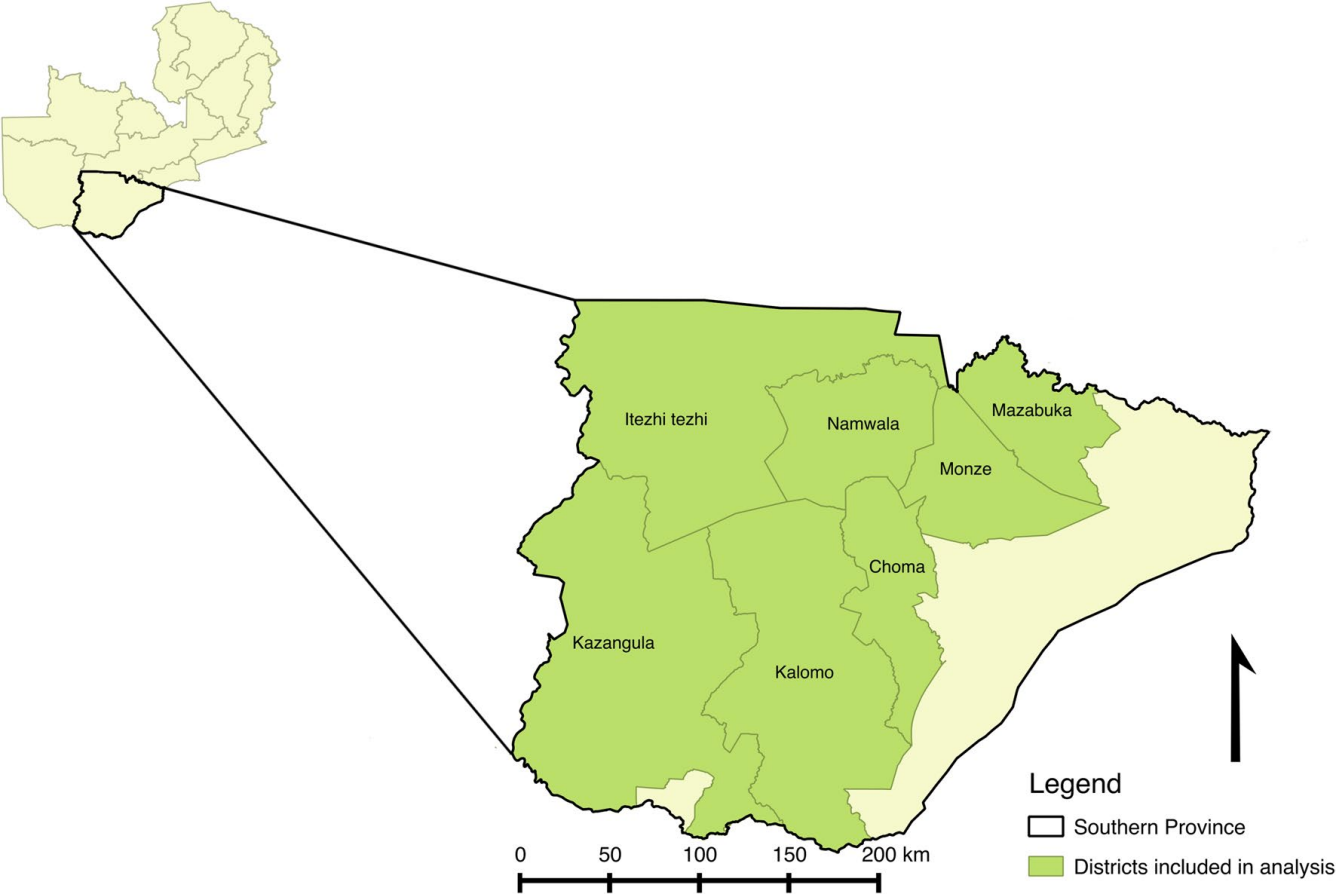
Districts in Zambia included in the analysis

This study took place in 7 districts in Southern Province, Zambia (Fig. 1). Malaria transmission in Southern Province is presumed to be low—malaria indicator surveys (MIS) conducted in 2008, 2010, 2012 and 2015 found parasite prevalence in children to be < 10% each year and large declines in malaria-related costs have been observed at two major hospitals [18]. The primary malaria vectors in Southern Province are *Anopheles arabiensis* and *Anopheles funestus.* Indoor residual spraying in the province is conducted annually although the proportion houses sprayed across the province is typically less than 30%, with many of the IRS resources targeted toward higher transmission districts bordering Lake Kariba (not included in this analysis). High coverage of insecticide-treated mosquito nets is observed (63.6% of households reporting at least one LLIN) and nets have been the mainstay of malaria prevention in the mostly rural areas where malaria is most prevalent [19].

### CCM and RCD system

Starting in 2011, the Government of the Republic of Zambia (GRZ) and partners implemented a CCM and RCD system in Southern Province, Zambia. This consisted of passive malaria CCM testing and treatment at the CHW health post (typically their household) and then in response to the identification of a positive case, RCD activities within 140 m of the incident case household [16]. Incident cases identified at health facilities also received RCD follow-up. By the end of 2014, a total of 1481 CHWs had been trained throughout Southern, Central and Western Provinces. CHWs conducted RCD as volunteers, although they did receive cell phone talk time and bicycles in remuneration for submitting monthly reports to the Health management information system (HMIS) via a DHIS2 (District Health Information Software 2) mobile-to-web system. Testing by CHWs is done with malaria rapid diagnostic tests, usually Standard Diagnostics SD Bioline^®^, and treatment for those testing positive follows the national malaria treatment guidelines, the majority of whom receive artemether-lumefantrine (AL), the first-line drug for uncomplicated malaria.

### Data

Monthly health facility data were retrieved from the National Malaria Elimination Centre (NMEC) surveillance database (which runs DHIS2) from January 2010 through December 2013. Reported malaria data for Zambia currently divides its databases into the standard health facility data (HMIS) and the expanded community-based data. Specifically, the following data elements were retrieved from the standard health facility database: total outpatient attendance, number of clinical malaria cases (those not confirmed by diagnostic), number of malaria diagnostic tests performed, number of confirmed malaria cases. The following data elements were retrieved from the community database: number of individuals seeking treatment from the CHW, number of suspected malaria cases tested for malaria, number of confirmed malaria cases, number of people tested for a malaria infection during case investigations, and number of people testing positive for a malaria infection during case investigations.

Monthly enhanced vegetation index and temperature were retrieved from the MODerate resolution Imaging Spectrometer (MODIS) satellite mission. Mean monthly values of EVI and temperature for areas within a 5 km buffer of each health centre were extracted and included in the study. Values were extracted using the raster package [20, 21] in R version 3.1.0 [22]. Monthly EVI and temperature were tested for time lags at 1 and 2 months, and a lag of 1 month was selected.

### Analyses

Due to known challenges with routinely reported malaria data [23], reporting completeness was measured by the proportion of facilities with non-missing values of malaria cases and non-zero values of outpatient attendance. Two separate analyses were conducted, one for the entire time period 2010–2013 and one for the time period 2012–2013 when data completeness was above 60%. Two separate groups of outcomes were considered, namely confirmed malaria infections (outcome A) and total outpatient attendance (outcome B). The outcome of confirmed malaria infections (outcome A) addresses the question of whether the CCM and RCD systems have increased access to diagnosis and treatment for malaria cases. A random effects negative binomial regression was applied to monthly confirmed malaria cases with the total outpatient attendance included as the offset to account for variations in treatment-seeking behaviour. A sinusoidal function of time was used to account for the seasonality of malaria, and additionally tested to determine if malaria infections increased in time with a categorical year covariate. EVI and temperature (lagged 1 month) were categorized as above or below the median and included as environmental factors. The type of facility (hospital, rural health centre, urban health centre and health post) was also included. These analyses were conducted considering two specific outcomes:

1. The number of confirmed malaria cases identified at the health facility.
2. The number of confirmed malaria cases identified at the health facility and by CHWs, i.e., including both CCM and RCD.

The above outcomes allow us to determine whether community activities have affected malaria incidence at the health facility (A.1), and/or whether there has been any change in treatment access due to the community-led efforts (A.2).

The outcome of total outpatient attendance (outcome B) addresses the question of whether the CCM and RCD systems have decreased the outpatient attendance at the health centre. A random effects linear regression was conducted of a log-transformed monthly total outpatient attendance at the health centre with the previously stated covariates included. This analysis was conducted with two specific outcomes:

1. The total outpatient attendance at health facilities only.
2. The total outpatient attendance at health facilities combined with patients seen by CHWs in the community.

Outcome B.1 gives a measure of whether the community activities have decreased the outpatient attendance at the health centre. Outcome B.2 gives a measure of how the community efforts expanded treatment-seeking behaviour or whether it is just shifted from health centre to the community. All analyses were conducted in Stata version 13.1 (Stata Corporation, College Station, Texas, USA).

## Results

**Fig. 2:**
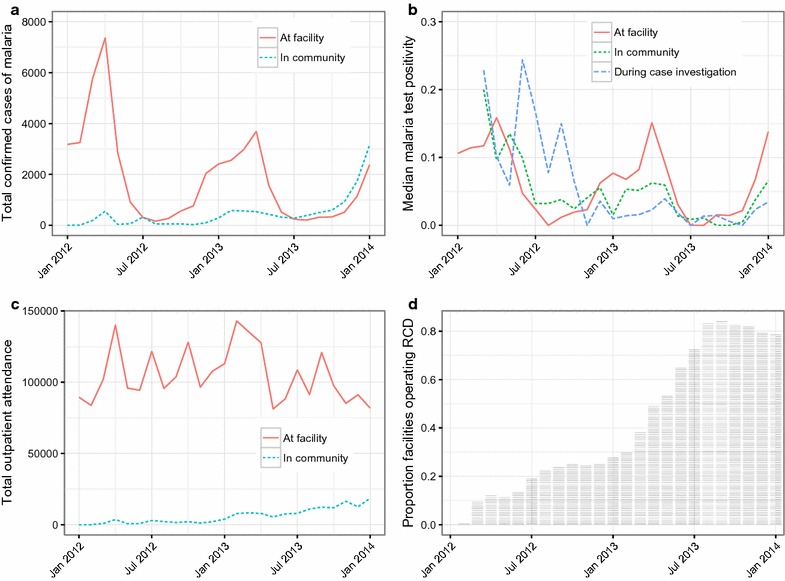
Monthly confirmed malaria cases (**a**), malaria test positivity (**b**), outpatient attendance (**c**) and implementation progress of community extension (**d**) at health centers included in the analysis

The increased support for malaria surveillance efforts began in 2011 in Southern Province and reporting completeness reached 60% in 2012 and remained at least at this level throughout 2013. Malaria test positivity was unavailable before 2012 and once measured was found to be < 20% throughout the study period, and < 10% in the dry season (June–December). Figure 2 shows trends of confirmed malaria cases, malaria test positivity and outpatient attendance by month 2012–2013. RCD scaled gradually in the study area beginning in 2012 to reach approximately 80% of health centres engaging in the interventions in 2013 (Fig. 2d).

### Confirmed malaria infections

Months when CHWs reported any malaria testing or malaria cases, an indication of when they were out in the community conducting malaria case management was not associated with malaria incidence as measured at the health facility when not taking into account the infections found in the community (p = 0.394, Table 1). However, once the malaria cases found in the community through CCM and RCD were combined with the health facility incidence, community efforts were associated with a 52% increase in confirmed malaria infections standardized to total outpatient attendance (incidence rate ratio [IRR] = 1.52, 95% confidence interval [CI] = 1.37–1.68). CHW health posts saw more malaria infections relative to outpatient attendance than rural health centres (IRR = 1.37, 95% CI = 1.05–1.79, Table 2). Environmental factors and seasonality were associated with increased confirmed malaria infections as expected (Table 2).

**Table 1:** Multivariate negative binomial regression analyses showing the relationship between confirmed malaria infections and the presence of CCM and RCD interventions for 2012–2013. Results for entire time period (2010-2013) available in additional file 1

Outcome	N observations (n health centers)	Incident rate ratio of any CCM alone (95% confidence interval)	Incident rate ratio of any RCD alone (95% confidence interval)	Incident rate ratio of any CCM+ RCD (95% confidence interval)
Health facility incidence only	3273 (137)	0.953 (0.852–1.065)	1.144 (1.038–1.262)**	0.953 (0.852–1.065)
Health facility incidence including community cases	2875 (137)	1.314 (1.186–1.457)***	1.397 (1.274–1.532)***	1.516 (1.365–1.683)***

Models controlled for malaria testing rate, type of facility, environmental factors as well as time and seasonality with a sinusoidal function

* p < 0.05, ** p < 0.01, *** p < 0.001

**Table 2:** Multivariate linear regression analysis showing the relationship between outpatient attendance and the presence of CCM and RCD interventions for 2012–2013

Factor	Categorization	Percent change (95% confidence interval)	p value
Community malaria treatment	Any CCM or RCD	− 5.99% (− 10.62 to 1.37%)	0.011
District	Choma	Reference	Reference
	Itezhi-tezhi	− 42.74% (− 92.23 to 6.75%)	0.091
	Kalomo	− 33.38% (− 64.63 to − 2.13%)	0.036
	Kazungula	− 26.43% (− 64.00 to 11.13%)	0.168
	Mazabuka	− 53.58% (− 87.90 to 19.26%)	0.002
	Monze	− 6.66% (− 39.68 to 26.36%)	0.693
	Namwala	37.24% (− 4.53 to 79.01%)	0.081
Year	2012	Reference	Reference
	2013	24.29% (15.60 to 34.98%)	< 0.001
Sinetime		26.07% (15.25 to 36.89%)	< 0.001
Costime		− 8.41% (− 19.43 to 2.61%)	0.135
Type of health centre	Rural health centre	Reference	Reference
	Hospital affiliated health centre	− 67.23% (− 125.6 to − 8.88%)	0.024
	Hospital	− 56.22% (− 113.3 to 0.87%)	0.054
	Health post	− 53.04% (− 82.72 to − 23.36%)	< 0.001
	Urban health centre	119.1% (81.79 to 156.4%)	< 0.001
Enhanced vegetation index	Below median	Reference	Reference
	Above median	− 5.97% (− 11.10–− 0.85%)	0.022
Monthly maximum daytime temperature	Below median	Reference	Reference
	Above median	− 0.86% (− 5.94 to 4.23%)	0.741
Monthly maximum nighttime temperature	Below median	Reference	Reference
	Above median	10.06% (6.62 to 13.50%)	< 0.001
Altitude	Below median	Reference	Reference
	Above median	25.79% (− 0.02 to 51.60%)	0.050

N = 2978 observations, 138 health centres

### Outpatient attendance

Months when CHWs reported any malaria testing or malaria cases for CCM and RCD were associated with a 6% decrease in the total outpatient attendance at the health facility (95% CI = 1.4–10.6% decrease, Table 3). When combining the outpatient attendance at the health facility with the outpatient attendance seen during CCM and people tested during RCD, however, there was no association (Table 3). Urban health centres had more than double the outpatient attendance compared to rural health centres (119% more, 95% CI = 82–156% more, Table 4), and community health posts had 53% fewer outpatients than rural health centres (95% CI = 23–83% fewer, Table 4). Environmental factors and seasonality were associated with outpatient attendance as expected (Table 4).

**Table 3:** Multivariate linear regression analyses showing the relationship between outpatient attendance and the presence of CCM and RCD interventions for 2012–13. Results for entire time period (2010-2013) available in additional file 1

Outcome	N observations (n health centres)	Percent change CCM alone (95% confidence interval)	Percent change RCD alone (95% confidence interval)	Percent change CCM + RCD (95% confidence interval)
Log-transformed outpatient attendance measured at health facility	3089 (138)	− 5.99% (− 10.62 to − 1.37%)*	0.59% (− 3.31–4.48%)	− 5.99% (− 10.62 to − 1.37%)*
Log-transformed outpatient attendance at health facility including individuals presenting and/or tested in community	3232 (138)	− 7.72% (− 14.09 to − 1.35%)*	3.67% (− 1.84–9.18%)	2.18% (− 4.14 to 8.50%)

Models controlled for type of facility, environmental factors as well as time and seasonality with a sinusoidal function

* p < 0.05

**Table 4:** Multivariate linear regression analysis showing the relationship between outpatient attendance and the presence of CCM and RCD interventions for 2012–2013

Factor	Categorization	Percent change (95% confidence interval)	p value
Community malaria treatment	Any CCM or RCD	− 5.99% (− 10.62 to 1.37%)	0.011
District	Choma	Reference	Reference
	Itezhi-tezhi	− 42.74% (− 92.23 to 6.75%)	0.091
	Kalomo	− 33.38% (− 64.63 to − 2.13%)	0.036
	Kazungula	− 26.43% (− 64.00 to 11.13%)	0.168
	Mazabuka	− 53.58% (− 87.90 to 19.26%)	0.002
	Monze	− 6.66% (− 39.68 to 26.36%)	0.693
	Namwala	37.24% (− 4.53 to 79.01%)	0.081
Year	2012	Reference	Reference
	2013	24.29% (15.60 to 34.98%)	< 0.001
Sinetime		26.07% (15.25 to 36.89%)	< 0.001
Costime		− 8.41% (− 19.43 to 2.61%)	0.135
Type of health centre	Rural health centre	Reference	Reference
	Hospital affiliated health centre	− 67.23% (− 125.6 to − 8.88%)	0.024
	Hospital	− 56.22% (− 113.3 to 0.87%)	0.054
	Health post	− 53.04% (− 82.72 to − 23.36%)	< 0.001
	Urban health centre	119.1% (81.79 to 156.4%)	< 0.001
Enhanced vegetation index	Below median	Reference	Reference
	Above median	− 5.97% (− 11.10 to − 0.85%)	0.022
Monthly maximum daytime temperature	Below median	Reference	Reference
	Above median	− 0.86% (− 5.94 to 4.23%)	0.741
Monthly maximum nighttime temperature	Below median	Reference	Reference
	Above median	10.06% (6.62 to 13.50%)	< 0.001
Altitude	Below median	Reference	Reference
	Above median	25.79% (− 0.02 to 51.60%)	0.050

N = 2978 observations, 138 health centres

## Discussion

This is the first assessment of which we are aware examining how CHWs conducting CCM and RCD may be affecting the broader health system. These data suggest that CHWs performing malaria CCM and RCD not only expand access to malaria treatment but also shift a significant portion of the malaria case management case load to CHWs. Months where CHWs reported conducting CCM and RCD were associated with a 50% increase in confirmed malaria infections after accounting for temporal and seasonal trends. This increase likely reflects an improvement in the capacity of malaria surveillance rather than an actual rise in malaria transmission as test positivity rates did not appear to increase during the time period included in this analysis (Fig. 2b). Further supporting this argument is the fact that malaria incidence measured at the health centre is only a fraction of the malaria burden [24]. By equipping CHWs to conduct CCM and RCD, the surveillance ‘net’ is wider, more granular and more sensitive and is thus able to detect more malaria infections than a surveillance system consisting of health facilities only. Improving the quality and capacity of a surveillance system in the context of malaria elimination is especially important; maximizing the number of malaria infections detected and treated will maximize the probability of eliminating existing parasite reservoirs, identifying importation and stopping onward transmission [25]. Similarly an improved surveillance system is crucial for documenting the maintenance of zero transmission and ultimately qualifying for WHO elimination certification. CCM alone did not provide as much benefit as RCD alone, and malaria elimination programmes should examine the feasibility of conducting RCD to improve surveillance.

Interestingly, the gains in malaria surveillance were made only when taking into account the malaria infections found during CCM and RCD. This strongly suggests that the CCM with RCD system that was implemented in Southern Province provides a tangible benefit in pursuing malaria elimination. Contrary to these findings other studies have found little benefit to RCD [26]. One of the main criticisms of RCD is that the RDTs currently used have difficulty detecting subclinical infections. Certainly RCD and malaria surveillance in general would benefit from improved diagnostics to identify asymptomatic infections, however these analyses suggests CCM and RCD provides a benefit with the current tools available.

Not only have community efforts improved access to malaria treatment, disaggregating malaria surveillance at the health post level improves the understanding of heterogeneity of malaria transmission within health centre catchment areas. The spatial refinement of surveillance is an important precursor to targeting malaria interventions such as indoor residual spraying, larviciding and/or insecticide-treated mosquito net distributions to geographical areas within health centre catchments.

Months when CHWs reported being in the community doing CCM and RCD were associated with a 6% decrease in the total outpatient attendance at the health facility. There is reason to believe that the decrease in outpatient attendance is likely due to fewer individuals with fever seeking care at health facilities. With a mean monthly outpatient attendance of 823 (95% CI: 798–848), a 6% decrease translates to roughly 49 fewer outpatients received at each health centre per month. Assuming each outpatient takes 10 min of time from healthcare providers at the health centre, those 49 fewer outpatients per month leads to approximately 8 person-hours of time saved per health centre per month when CHWs are conducting malaria community case management. The decrease in outpatient attendance and person-hours saved at the health centre did not account for increased time spent by CHWs conducting RCD. Volunteer CHWs could potentially become overburdened by RCD, particularly in areas where malaria transmission is more intense. When people being tested for malaria during CCM and RCD were included in the measure of outpatient attendance, there was only an association for CCM suggesting that the lower attendance may be due to individuals seeking and receiving care in the community rather than going to the health facility.

These analyses have some limitations. Firstly, it should be noted that the use of IRR in the analysis is due to the negative binomial regression model, but the incidence also includes prevalence cases found through RCD. Second, routinely reported malaria case data often has inherent problems; reporting completeness and treatment-seeking behaviour are often noted as most problematic [23]. To mitigate the issue of reporting completeness, we measured the level of reporting completeness and conducted analyses on time periods with at least 60% reporting completeness. To account for the issue of treatment-seeking behaviour at health facilities, confirmed malaria infections standardized to total outpatient attendance. Including a measure of time also helped to account for variation in treatment-seeking behaviour due to seasonality. A second limitation of this analysis was that not all suspected malaria cases are confirmed at health centres using either a malaria rapid diagnostic test or microscopy. This issue was addressed by including malaria testing rate as a covariate in the analyses of confirmed malaria infections to account for those facilities that would appear to have higher confirmed malaria infections, but in reality just have higher testing rates. Finally, the interventions were scaled gradually, limiting the ability to do a more robust analysis that might incorporate a time by intervention interaction. The associations seen here may perhaps be caused by temporal drift including varying coverage and/or effectiveness of vector control interventions such as ITNs, though measures of time were incorporated through the use of a sinusoidal function and categorical year. Despite these limitations, these findings strongly support the case for implementing community-led malaria surveillance and interventions.

## Conclusions

Community implemented CCM and RCD are associated with decreased outpatient attendance at health centres and increased capacity of malaria surveillance systems, respectively. Scaling these programmes will likely lead to tangible benefits for both targeting malaria specific interventions and resources to areas with higher case loads and expanding the capacity of a currently overburdened health system. Augmenting the CHW skillset with the ability to treat diarrhea and pneumonia would likely lead to even larger gains in terms of expanding the access to care, easing the burden on health facilities, and ensuring continued use of community-based care in a malaria elimination environment. These gains need to be weighed against the additional workload placed on volunteer CHWs.

## Additional file

**Additional file 1.** Additional tables showing the analyses for the entire time period (2010–2013).

## Abbreviations

AL: artemether-lumefantrine
CHW: community health worker
CI: confidence interval
CCM: community case management
DHIS2: District Health Information System 2
GRZ: Government of the Republic of Zambia
HMIS: health management information system
IRR: incident rate ratio
MACEPA: the Malaria Control and Elimination Partnership in Africa, a program at PATH
MIS: malaria indicator survey
MODIS: moderate resolution imaging spectrometer
NMEC: National Malaria Elimination Centre
RCD: reactive case detection

## References

[1] Black RE, Cousens S, Johnson HL, Lawn JE, Rudan I, Bassani DG (2010). Global, regional, and national causes of child mortality in 2008: a systematic analysis. Lancet.

[2] Schellenberg JA, Newell JN, Snow RW, Mung’ala V, Marsh K, Smith PG (1998). An analysis of the geographical distribution of severe malaria in children in Kilifi District, Kenya. Int J Epidemiol.

[3] Schellenberg, Victoria CG, Mushi A, De Savigny D, Scellenberg D, Mshinda H (2003). Inequities among the very poor: health care for children in rural southern Tanzania. Lancet.

[4] Littrell M, Miller JM, Ndhlovu M, Hamainza B, Hawela M, Kamuliwo M (2013). Documenting malaria case management coverage in Zambia: a systems effectiveness approach. Malar J.

[5] Hopkins H, Talisuna A, Whitty CJ, Staedke SG (2007). Impact of home-based management of malaria on health outcomes in Africa: a systematic review of the evidence. Malar J.

[6] Travis P, Bennett S, Haines A, Pang T, Bhutta Z, Hyder AA (2004). Overcoming health-systems constraints to achieve the Millennium Development Goals. Lancet.

[7] Rao VB, Schellenberg D, Ghani AC (2013). Overcoming health systems barriers to successful malaria treatment. Trends Parasitol.

[8] Stratton L, O’Neill MS, Kruk ME, Bell ML (2008). The persistent problem of malaria: addressing the fundamental causes of a global killer. Soc Sci Med.

[9] Collins FS, Glass RI, Whitescarver J, Wakefield M, Goosby EP (2010). Developing health workforce capacity in Africa. Science.

[10] Chen L, Evans T, Anand S, Boufford JI, Brown H, Chowdhury M (2004). Human resources for health: overcoming the crisis. Lancet.

[11] Schatz JJ (2008). Zambia’s health-worker crisis. Lancet.

[12] Gow J, George G, Mutinta G, Mwamba S, Ingombe L (2011). Health worker shortages in Zambia: an assessment of government responses. J Public Health Policy.

[13] Christopher JB, Le May A, Lewin S, Ross DA (2011). Thirty years after Alma-Ata: a systematic review of the impact of community health workers delivering curative interventions against malaria, pneumonia and diarrhoea on child mortality and morbidity in sub-Saharan Africa. Hum Resour Health.

[14] Chanda P, Hamainza B, Moonga HB, Chalwe V, Pagnoni F (2011). Community case management of malaria using ACT and RDT in two districts in Zambia: achieving high adherence to test results using community health workers. Malar J.

[15] Yeboah-Antwi K, Pilingana P, Macleod WB, Semrau K, Siazeele K, Kalesha P (2010). Community case management of fever due to malaria and pneumonia in children under five in Zambia: a cluster randomized controlled trial. PLoS Med.

[16] Larsen DA, Chisha Z, Winters B, Mwanza M, Kamuliwo M, Mbwili C (2015). Malaria surveillance in low-transmission areas of Zambia using reactive case detection. Malar J.

[17] Stresman GH, Kamanga A, Moono P, Hamapumbu H, Mharakurwa S, Kobayashi T (2010). A method of active case detection to target reservoirs of asymptomatic malaria and gametocyte carriers in a rural area in Southern Province, Zambia. Malar J.

[18] Comfort AB, van Dijk JH, Mharakurwa S, Stillman K, Gabert R, Korde S (2014). Hospitalizations and costs incurred at the facility level after scale-up of malaria control: pre-post comparisons from two hospitals in Zambia. Am J Trop Med Hyg.

[19] Chizema-Kawesha E, Miller JM, Steketee RW, Mukonka VM, Mukuka C, Mohamed AD (2010). Scaling up malaria control in Zambia: progress and impact 2005–2008. Am J Trop Med Hyg.

[20] Hijmans RJ, van Etten J. raster: Geographic Analysis and Modeling with Raster data. R package version. 2012.

[21] Hijmans RJ. Introduction to the’raster’package (version 2.0-08). 2012.

[22] Team RDC. R: A language and environment for statistical computing. http://www.R-project.org/. R foundation for statistical computing; 2010.

[23] Rowe AK, Kachur SP, Yoon SS, Lynch M, Slutsker L, Steketee RW (2009). Caution is required when using health facility-based data to evaluate the health impact of malaria control efforts in Africa. Malar J.

[24] Breman JG (2001). The ears of the hippopotamus: manifestations, determinants, and estimates of the malaria burden. Am J Trop Med Hyg.

[25] Barclay VC, Smith RA, Findeis JL (2012). Surveillance considerations for malaria elimination. Malar J.

[26] van Eijk AM, Ramanathapuram L, Sutton PL, Kanagaraj D, Sri Lakshmi Priya G, Ravishankaran S (2016). What is the value of reactive case detection in malaria control? A case-study in India and a systematic review. Malar J.

