# Supplementary material for: Shifting the burden or expanding access to care? Assessing malaria trends following scale-up of community health worker malaria case management and reactive case detection
Source: Malar J. 2017 Nov 2;16:441. doi: 10.1186/s12936-017-2088-1 (PMC5668974; doi:10.1186/s12936-017-2088-1)
Supplement: Supplementary file 1 — Additional file 1. Additional tables showing the analyses for the entire time period (2010–2013). [file 12936_2017_2088_MOESM1_ESM.docx]

Supplemental table 1. Multivariate negative binomial regression analyses showing the relationship between confirmed malaria infections and the presence of CCM and RCD interventions for 2010-13. Models controlled for malaria testing rate, year, type of facility, environmental factors as well as time and seasonality with a sinusoidal function.

| Outcome | N observations (n health centers) | Incident rate ratio of any CCM alone (95% Confidence interval) | Incident rate ratio of any RCD alone (95% Confidence interval) | Incident rate ratio of any CCM + RCD (95% Confidence interval) |
| --- | --- | --- | --- | --- |
| Health facility incidence only | 3273 (137) | 1.002 (0.904 – 1.112) | 1.163 (1.061 – 1.276)** | 1.002 (0.0.904 – 1.112) |
| Health facility incidence including community cases | 3282 (137) | 1.376 (1.249 – 1.516)*** | 1.405 (1.287 – 1.535)*** | 1.579 (1.432 – 1.742)*** |

*p < 0.05 **p < 0.01 *** p < 0.001

Supplemental table 2. Multivariate linear regression analyses showing the relationship between outpatient attendance and the presence of CCM and RCD interventions for 2010-13. Models controlled for type of facility, environmental factors as well as time and seasonality with a sinusoidal function and a year covariate.

| Outcome | N observations (n health centers) | Percent change CCM alone (95% confidence interval) | | Percent change RCD alone (95% confidence interval) | Percent change CCM +RCD (95% confidence interval) |
| --- | --- | --- | --- | --- | --- |
| Log-transformed outpatient attendance measured at health facility | 3500 (138) | -6.99% (-11.56 - -2.41%)** | -0.77% (-4.75 – 3.20%) | | -6.99% (-11.56 - -2.41%)** |
| Log-transformed outpatient attendance at health facility including individuals presenting and/or tested in community | 3648 (138) | -9.55% (-15.56 - -3.54%)** | 1.80% (-3.73 – 7.33%) | | -1.51 % (-7.61 – 4.58%) |

** p < 0.01

Supplemental table 3. Multivariate linear regression analysis showing the relationship between outpatient attendance and the presence of CCM and RCD interventions for 2012-2013. N = 2978 observations, 138 health centers

| Factor | Categorization | Percent change (95% confidence interval) | P-value |
| --- | --- | --- | --- |
| Community malaria treatment | Any CCM or RCD | -5.99% (-10.62 – 1.37%) | 0.011 |
| District | Choma | Reference | Reference |
|  | Itezhi-tezhi | -42.74% (-92.23 – 6.75%) | 0.091 |
|  | Kalomo | -33.38% (-64.63 - -2.13%) | 0.036 |
|  | Kazungula | -26.43% (-64.00 – 11.13%) | 0.168 |
|  | Mazabuka | -53.58% (-87.90 – 19.26%) | 0.002 |
|  | Monze | -6.66% (-39.68 – 26.36%) | 0.693 |
|  | Namwala | 37.24% (-4.53 – 79.01%) | 0.081 |
| Year | 2012 | Reference | Reference |
|  | 2013 | 24.29% (15.60 – 34.98%) | <0.001 |
| Sinetime |  | 26.07% (15.25 – 36.89%) | <0.001 |
| Costime |  | -8.41% (-19.43 – 2.61%) | 0.135 |
| Type of health center | Rural health center | Reference | Reference |
|  | Hospital affiliated health center | -67.23% (-125.6 - -8.88%) | 0.024 |
|  | Hospital | -56.22% (-113.3 – 0.87%) | 0.054 |
|  | Health post | -53.04% (-82.72 - -23.36%) | <0.001 |
|  | Urban health center | 119.1% (81.79 – 156.4%) | <0.001 |
| Enhanced vegetation index | Below median | Reference | Reference |
|  | Above median | -5.97% (-11.10 - -0.85%) | 0.022 |
| Monthly maximum daytime temperature | Below median | Reference | Reference |
|  | Above median | -0.86% (-5.94 – 4.23%) | 0.741 |
| Monthly maximum nighttime temperature | Below median | Reference | Reference |
|  | Above median | 10.06% (6.62 – 13.50%) | <0.001 |
| Altitude | Below median | Reference | Reference |
|  | Above median | 25.79% (-0.02 – 51.60%) | 0.050 |

Supplemental table 4. Multivariate negative binomial regression analysis showing the relationship between confirmed malaria infections found anywhere (health center, CCM or RCD) and the presence of CCM or RCD interventions for 2012-13. Model includes health center as a random intercept and is standardized to total outpatient attendance (a combination of outpatient attendance at health center, outpatient attendance during CCM and people tested during RCD). N = 2786 observations, 137 health centers.

| Factor | Categorization | Incident rate ratio (95% confidence interval) | P-value |
| --- | --- | --- | --- |
| Community malaria treatment | Any CCM or RCD | 1.516 (1.365 – 1.683) | <0.001 |
| Testing rate |  | 4.783 (3.659 – 6.252) | <0.001 |
| District | Choma | Reference | Reference |
|  | Itezhi-tezhi | 1.493 (1.036 – 2.151) | 0.032 |
|  | Kalomo | 1.313 (1.057 – 1.632) | 0.014 |
|  | Kazungula | 0.924 (0.697 – 1.224) | 0.580 |
|  | Mazabuka | 1.077 (0.802 – 1.448) | 0.621 |
|  | Monze | 1.134 (0.886 – 1.452) | 0.318 |
|  | Namwala | 0.352 (0.252 – 0.493) | <0.001 |
| Year | 2012 | Reference | Reference |
|  | 2013 | 0.852 (0.707 – 1.025) | 0.090 |
| Sinetime |  | 0.968 (0.788 – 1.188) | 0.754 |
| Costime |  | 1.599 (1.268 – 2.017) | <0.001 |
| Type of health center | Rural health center | Reference | Reference |
|  | Hospital affiliated health center | 1.810 (1.052 – 3.115) | 0.032 |
|  | Hospital | 1.209 (0.739 – 1.976) | 0.449 |
|  | Health post | 1.368 (1.046 – 1.789) | 0.022 |
|  | Urban health center | 0.774 (0.596 – 1.006) | 0.056 |
| Enhanced vegetation index | Below median | Reference | Reference |
|  | Above median | 2.339 (2.108 – 2.594) | <0.001 |
| Monthly maximum daytime temperature | Below median | Reference | Reference |
|  | Above median | 0.703 (0.635 – 0.778) | <0.001 |
| Monthly maximum nighttime temperature | Below median | Reference | Reference |
|  | Above median | 1.115 (1.033 – 1.202) | 0.005 |
| Altitude | Below median | Reference | Reference |
|  | Above median | 1.260 (1.026 – 1.546) | 0.027 |
